# The impact of land-use intensity on the community dynamics of colonial volvocine algae in the Yangtze River Basin

**DOI:** 10.1128/spectrum.02174-25

**Published:** 2025-12-17

**Authors:** Yuxin Hu, Jiwei Zhang, JingJing Lin, Xiaolong Huang, Jie Huang

**Affiliations:** 1 Changjiang Basin Ecology and Environment Monitoring and Scientific Research Center, Changjiang Basin Ecology and Environment Administration, Ministry of Ecology and Environment, Wuhan, Hubei, China; 2 Hubei Provincial Key Laboratory for Basin Ecology Intelligent Monitoring-Prediction and Protection, Wuhan, China; Institute of Eco-environmental and Soil Sciences, Guangdong Academy of Sciences, Guangzhou, Guangdong, China

**Keywords:** land-use, colonial volvocine algae, community composition, community assembly, homogenization, Yangtze River basin

## Abstract

Colonial volvocine algae (CVA), which range from four-celled to complex multicellular forms, are vital for understanding life evolution. This study aims to quantify the impacts of human land-use intensity on CVA communities across the Yangtze River Basin and explore how anthropogenic changes drive CVA ecological niche dynamics and assembly processes. Findings reveal that increased land-use intensity reduces forest cover, expands construction land, and elevates water nutrients, thereby worsening water quality. This environmental shift provides elevated nutrient resources for CVA, expanding their ecological niche width (*P* < 0.01) and reducing competition (interspecific niche overlap network simplified), particularly benefiting larger-bodied colonial volvocine algae, such as *Volvox carteri*. However, morphological similarity among CVA species, such as *Eudorina elegans* and *Colemanosphaera charkowiensis*, results in niche overlap and intensified competition. Notably, CVA complexity does not substantially correlate with distribution patterns, indicating that factors other than basic morphological characteristics play a role in determining their ecological success. On the other hand, CVA community assembly in the Yangtze River Basin, analogous to eukaryote and prokaryote, was initially dominated by stochastic processes at low land-use intensities (β-NTI mostly ranges between −2 and 2). Increased intensity enhances environmental filtering, leading to community homogenization (β-diversity decreased from 0.9119 ± 0.2236 under low HAILS to 0.8974 ± 0.2332 under high HAILS, *P* < 0.001), which diminishes biodiversity, alters ecological processes, and reduces ecological stability, ultimately affecting ecosystem integrity and sustainability. This study highlights that land-use intensification-induced environmental changes restructure CVA community dynamics, niche relationships, and assembly processes. These findings provide critical insights for sustainable land management and freshwater biodiversity conservation.

**IMPORTANCE:**

This study underscores the importance of colonial volvocine algae as a model for understanding how human land-use intensification reshapes freshwater biodiversity, ecological niches, and community assembly, offering key insights for sustainable ecosystem management.

## INTRODUCTION

Colonial volvocine algae (CVA), a diverse group of freshwater planktonic algae comprising approximately 60 species within the order Volvocales, encompassing simple to complex morphologies, are vital for understanding biological evolution (1–4). Investigating CVA ecology is vital for refining evolutionary theories, adapting to human impacts, and conserving biodiversity. Despite their simplicity-to-complexity spectrum (5), both systems can exhibit high fitness (6). Yet, the Red Queen Principle implies that constant change is necessary for maintaining adaptiveness (7), leading many systems to evolve with increased complexity (8). Notably, CVA’s gradient from simple to complex morphologies makes them an ideal system to test how complexity relates to adaptiveness. However, it remains unexplored which CVA species possess superior ecological adaptability, higher abundance, and a broader distribution.

Land-use changes have induced shifts in chemical conditions, evidenced by increased nutrients in water bodies, reduced dissolved oxygen levels, and degradation of water quality (9). These alterations can significantly impact phytoplankton populations and affect aquatic ecosystems (10, 11), with studies focusing on the effects of urban construction land, farmland, and natural land-use proportions and arrangements on phytoplankton (12–14). Findings indicate that urban land-use expansion, coupled with forest habitat reductions, can lead to higher phytoplankton densities in water bodies (13). Meanwhile, with human activities such as urbanization affecting CVA interspecies dynamics and community structure—potentially impacting aquatic ecosystem health (15)—understanding CVA’s environmental responses is essential for predicting and managing these human-induced effects.

Defined from the perspective of land use, Human Activity Intensity of Land Surface (HAILS) refers to the degree to which humans utilize, transform, and develop the natural surface cover within a specific area. Such a degree can be reflected by land use/cover types, thus acting as an indicator of land-use intensity (16). Notably, previous studies have demonstrated that increased land-use intensity—i.e., higher HAILS—strengthens the role of environmental filtering (environmental filtering refers to the process by which biotic and abiotic factors in an environment select for species, where only those with ecological traits matching local environmental conditions can survive, colonize, and reproduce in the habitat) in shaping prokaryotic community assembly (17). Building on this, to better understand how human activity influences eukaryotic community assembly, we now focus on the CVA group. The complexity of CVA organisms’ evolutionary structures improves their environmental adaptability. This aligns with niche theory, which holds that species traits and trade-offs determine how species use resources and differ in ecological niches, thereby allowing for stable coexistence (18, 19). Alternatively, the neutral theory suggests that community dynamics are random and driven by individual ecological drift (20, 21). However, the relative contributions of niche-based and neutral processes to CVA community assembly under varying land-use intensities remain unclear, limiting our ability to predict how human activities reshape these model communities.

Considering the mentioned backdrop, we applied metabarcoding technology to acquire community data on CVA through an investigation in the Yangtze River Basin. Our goal was to explore the following three scientific questions: (i) How does intensified human land use influence community dynamics and biodiversity among CVA? (ii) What effects do environmental alterations exert on the ecological niches and species interactions within CVA? (iii) As land-use intensity fluctuates, how do stochastic assembly processes and nutrient-driven environmental filtering shape CVA communities, and what consequences arise for community structure?

## MATERIALS AND METHODS

### Sampling and laboratory analysis

Spanning approximately 6,300 km and covering a basin area of 1.8 million km², the Yangtze is Asia’s largest river and the third longest in the world. The river’s basin harbors diverse ecosystems, from forests to coastlines, originating in the Tibetan Plateau’s glaciers and culminating in the East China Sea, supporting a wealth of biodiversity (22–24).

In this study, water samples were collected at 235 different locations throughout the basin from June to August 2021. 1.5 L of water was collected from each sampling location into a clean PET bottle. The samples were delivered to a neighboring laboratory within 2 h of collection and stored at 0–4°C upon arrival. 1.5 L of the stored water was filtered within 1 h of storage using 0.22 µm polycarbonate membranes (Millipore, United States). These filtration membranes were then frozen at −80°C until DNA extraction.

Using a portable GPS device (Magellan, United States), the longitude and latitude of each sampling location were recorded while in the field. Water quality parameters, such as total phosphorus (TP), total nitrogen (TN), ammonium nitrogen (AN), and chemical oxygen demand by potassium permanganate (COD), were measured according to the Water and Waste Water Monitoring and Analysis Standard Methods (Editorial Committee of Ministry of Environmental Protection of China, 2002). The other conventional water parameters, including dissolved oxygen (DO), water temperature (Temp), pH, and conductivity (Cond), were measured *in situ* by a portable multi-parameter water quality analyzer (YSI pro Quatro 24F10500, Xylem).

### Sequencing and bioinformatics analysis

Genomic DNA was extracted using a PowerSoil DNA Isolation Kit (MoBio) following the manufacturer’s instructions. Then, the full-length 18S ribosomal RNA gene was amplified by PCR using barcoded primers Euk-A (AACCTGGTTGATCCTGCCAGT) and Euk-B (GATCCTTCTGCAGGTTCACCTAC) (25, 26). The PCR reactions were carried out with polymerase (KOD FX Neo, Toyobo). The products were extracted from 2% agarose gels, purified using the Monarch DNA Gel Extraction Kit (New England Biolabs, Ipswich MA, United States), and quantified by Qubit (Thermo Scientific). Purified amplicons were pooled in equimolar amounts and sequenced on the PacBio Sequel II platform (PacBio, United States).

Raw reads were demultiplexed using lima v1.7.0 (github.com/pacificbiosciences/barcoding) based on barcode sequence, and then, the circular consensus sequence (CCS) reads were generated from the raw PacBio sequencing data by SMRT link v8 (https://www.pacb.com/products-and-services/analytical-software/smrt-analysis/). The CCS was analyzed using the R package DADA2 v1.22.0 (25), and the parameters were set according to the reference study (27). Specifically, the "removePrimers" function is utilized to eliminate primers and align CCS reads in the forward direction (orient = TRUE). The "filterAndTrim" function (minQ = 3, maxEE = 2, maxN = 0, rm.phix = TRUE) is employed for sequence filtration and trimming to get clean data. The "learnErrors" function is used to estimate the error model of the data. The "dada" function is applied to infer amplicon sequence variants (ASV). The "removeBimeraDenovo" function is used to eliminate chimeras (method="consensus"). Finally, the "assignTaxonomy" function and SILVA data set (silva_132.18s.99_rep_set.dada2.fa.gz, downloaded from https://zenodo.org/record/1447330 as of October 28, 2022) are used for species-level annotation. Quality-score plots of the raw data and clean data were conducted using Multiqc (28), revealing that the Phred scores for all sequences of clean data exceeded 80 (Fig. S1), thereby affirming the robust quality of the sequences.

The local database was constructed to ensure the accuracy of CVA annotations (29). Briefly, all colonial volvocine algae 18S rRNA sequences were downloaded from NCBI; short and redundant reads were filtered out; alignments and phylogenetic analyses were performed; and the resulting curated data set was used for BLAST-based annotation. For additional clarity on the implementation, all colonial volvocine algae sequences of 18S rRNA reads were downloaded from the NCBI Nucleotide database (https://www.ncbi.nlm.nih.gov/nuccore), and short reads (less than 1,500 bp) and redundant reads were eliminated using CD-HIT (30). Phylogenetic analysis was then performed using MAFFT v7.490 (31), trimAL v1.2 (32), and FastTree v2.1.11 (33), with incorrect reads being discarded. The sequences annotated to CVA were analyzed by BLASTn search with BLAST+ (34) against the local reference database at >98.5% similarity. The taxonomic information of each ASV was manually checked, resulting in the classification of 41 ASVs belonging to CVA. Finally, in consideration of the fact that most CVA are polyphyletic groups (2), we constructed trees for each genus separately according to the above phylogenetic analysis methods, and the phylogenetic results show that the identification of CVA at the species level is correct (Fig. S2 to S9).

### Land-use data

Land-use data (at a 30 m resolution) in the year 2021 were acquired according to the reference study (https://zenodo.org/records/8176941). The basin was divided into six primary land-use kinds—farmland, forest, grassland, natural water, unused land, and construction land—considering the features of this basin’s land cover to accurately assess the land-use structure. Five different-sized buffers were constructed: a 0.5 km, a 1 km, a 1.5 km, a 2 km, and a 2.5 km buffer. Redundancy analysis (RDA) and the Mantel test were used to examine the relation between land-use types and water quality parameters under different buffer scales by R package "vegan" v2.6.4. HAILS, which quantifies land-use intensification, was calculated as the proportion of construction land area to the total area within the 2,000 m buffer (consistent with subsequent RDA analysis), following the reference study (16).

### Data analysis

Water quality indices (WQI) were calculated by Temp, pH, Cond, DO, TN, AN, TP, and COD and merged into the final index (35). The geom_smooth (method = "lm") in R package "ggplot2" v3.4.2 was used to perform linear regression to examine the association between different water quality parameters, the area of different land-use types, and HAILS. The partial least squares discriminant analysis (PLS-DA) based on R packages "ropls" v1.26.4 and the analysis of similarities (ANOSIM) based on R packages "vegan" v2.6.4 are used to test statistically whether there is a significant difference between low HAILS and high HAILS. The "cor.test" function in R v4.1.2 was used to test for correlation between paired parameters. The Wilcoxon rank-sum test used in this study is based on the "geom_signif" function in R package "ggsignif" v0.6.4 with the option "test = wilcox.test". Based on the R package "spaa" v0.2.2, the niche width of CVA is calculated by the function "niche.width" with the option "method = levins", and the niche overlap between each pair of CVA is calculated by the function "niche.overlap" with the option "method = levins".

The ecological processes of CVA communities are identified by β nearest-taxon index (β-NTI) and modified Raup-Crick index (RCBray), which are calculated based on the "qpen" function in R package "iCAMP" v1.5.12 using the options "sig.bNTI=2" and "sig.rc=0.95" (36). The Bray–Curtis distance index was used to characterize the β-diversity, which is calculated based on the "vegdist" function in R package "vegan" v2.6.4.

The environmental parameter was transformed into a Euclidean distance matrix (based on the "vegdist" function in R package "vegan" v2.6.4 with option method = "euclidean"). Additionally, a spatial distance matrix between different CVA communities was calculated using latitude and longitude coordinates obtained through GPS and the "distm" function in the "geosphere" v1.5 R package. The relative importance of land use, environmental factors, and geodistance was further assessed using the variation partitioning analysis (VPA) by the "varpart4" function in the R script "RFunctions.R" (https://github.com/csdambros/BioGeoAmazonia), according to the previous study (17). Similarity percentage (SIMPER) procedures were applied to determine the characteristic species under different trophic gradients based on the "simper" function in R package "vegan" v2.6.4.

## RESULTS

### Characteristics of sampling sites with CVA distribution

**Fig 1:**
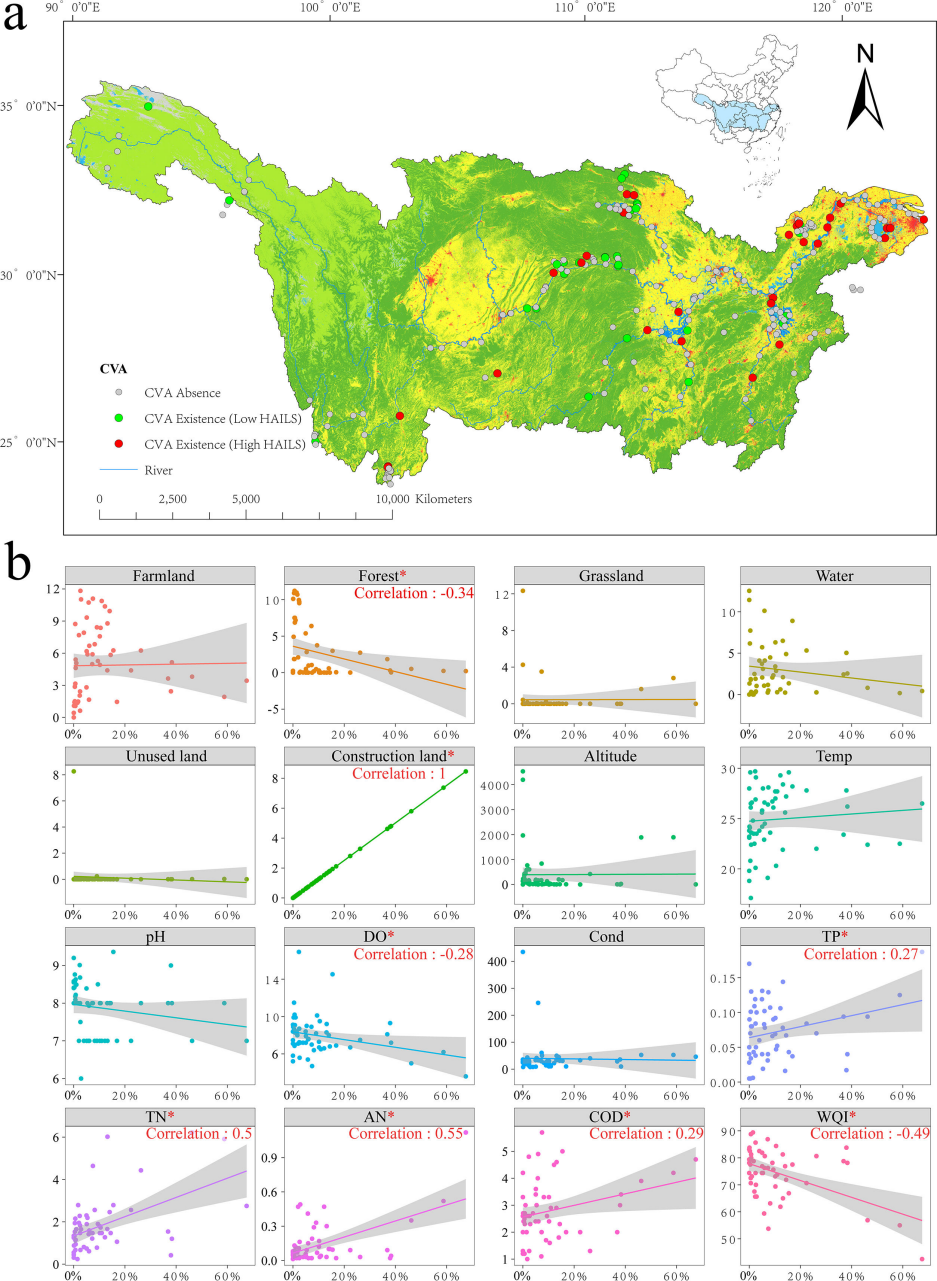
Study area, sampling sites, and land-use types in the Yangtze River Basin. (**a**) Land-use types are categorized as forest (dark green), cropland (beige), impervious surfaces (pink), water (blue), barren (brown), and grassland (light green) (37). Sampling sites are denoted by circles: gray circles represent locations where CVA is absent; green circles indicate sites with CVA existence under low HAILS; and red circles signify sites with CVA existence under high HAILS. (**b**) Linear regression between different land-use types and water quality parameters with HAILS. Variables marked with a red asterisk (*) indicate that the correlations are statistically significant (*P* < 0.05), and correlation coefficients are shown for these plots. The gray shaded areas around the trend lines in each scatter plot represent the confidence intervals.

Within the Yangtze River Basin, CVA was identified at 58 of 235 sampling sites (Fig. 1a). Buffer zones around these sites, ranging from 500 m to 2,500 m in radius, showed that as the radius increased, the proportion of farmland, forest, and grassland expanded, while that of natural water, construction land, and unused land decreased (Fig. S10). This reflects an urbanization gradient where central city areas are more developed, with a greater proportion of natural or semi-natural lands toward the periphery. Consequently, larger buffer zones often encompass more distant regions that are less affected by human activities and more naturally preserved.

RDA analysis across various buffer zones highlighted the highest explanatory rate at the 2,000 m buffer (Fig. S11), implying a significant influence of land use on water quality at this scale. Subsequent analyses thus focused on the 2,000 m buffer, where the Mantel test revealed a strong positive correlation between construction land and eutrophication indicators (TN, TP, AN) (Fig. S12), suggesting that expansion in construction land markedly increases water nutrient concentrations.

Linear regression further analyzed correlations with HAILS (Fig. 1b), revealing a significant negative association between forests and HAILS, indicating that forest area diminishes as HAILS increases. Additionally, DO and WQI decrease, while TP, TN, AN, and COD levels rise with higher HAILS, suggesting water quality deterioration and increased nutrient concentrations in the water environment.

### Impact of land-use intensification on CVA community composition and water quality

HAILS values were calculated within a 2,000 m buffer for each site, with sites categorized as high HAILS if their HAILS exceeded the median threshold and as low HAILS if not (Fig. 1a). PLS-DA analysis distinctly separated the two groups, and ANOSIM confirmed significant differences between these HAILS-based clusters (*P* < 0.05, Fig. S13).

**Fig 2:**
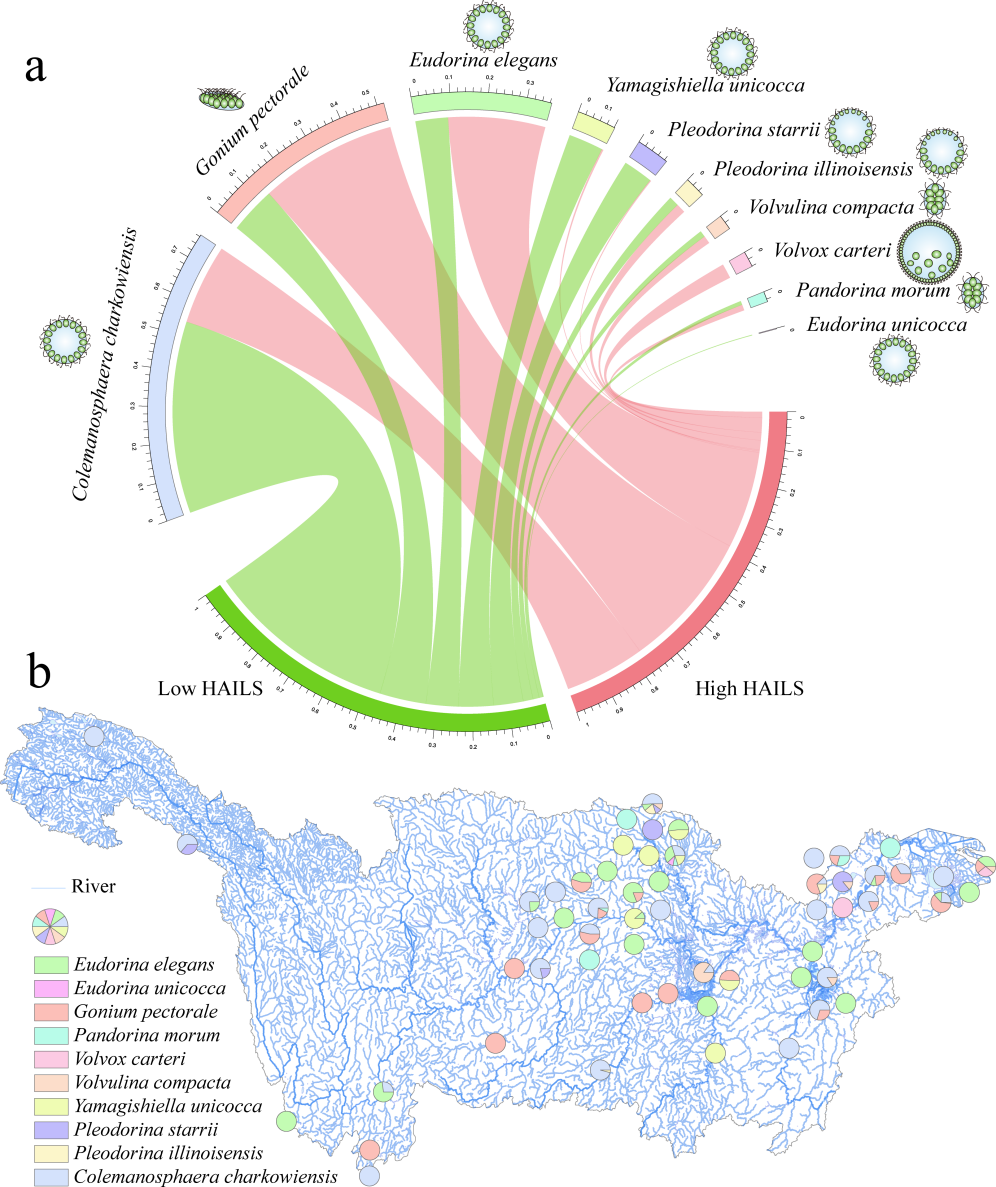
CVA community composition in the Yangtze River Basin. (**a**) Circular visualization of CVA community composition at species level in low and high HAILS; the inner circular diagram shows relative abundance of different species; and the width of ribbons for each species is directly proportional to their relative abundance in each HAILS. (**b**) CVA community composition at each sampling site (39). Colored pie charts at different locations represent the presence of specific algae. Each pie chart visually conveys the occurrence of these algal species within the sampled areas of the river system.

Our study identified 41 ASVs attributed to the CVA group, representing 2 families and 10 species. The number of CVA species and their distribution reported herein are generally consistent with those in previous research (38). Community structure analysis under varying HAILS (Fig. 2a) revealed higher relative abundances of *Colemanosphaera charkowiensis*, *Yamagishiella unicocca*, and *Pleodorina starrii* in low HAILS, whereas *Gonium pectorale*, *Eudorina elegans*, and *Volvox carteri* were more prevalent in high HAILS. In terms of distribution across sampling sites (Fig. 2b), *Colemanosphaera charkowiensis* was found at 27 sites, leading in widespread presence, followed by *Eudorina elegans* and *Gonium pectorale*. Conversely, *Volvox carteri*, the most complex within the CVA and characterized by complex colony morphology (hundreds of thousands of cells) and oogamous reproduction, was detected at only two sites. In contrast, *Tetrabaena socialis*, the simplest one with simple colony morphology (only four cells) and isogamous reproduction, was not observed at all. This suggests no significant correlation between CVA organism complexity (defined by colony cell number and reproductive mode) and their distribution in the Yangtze River Basin.

**Fig 3:**
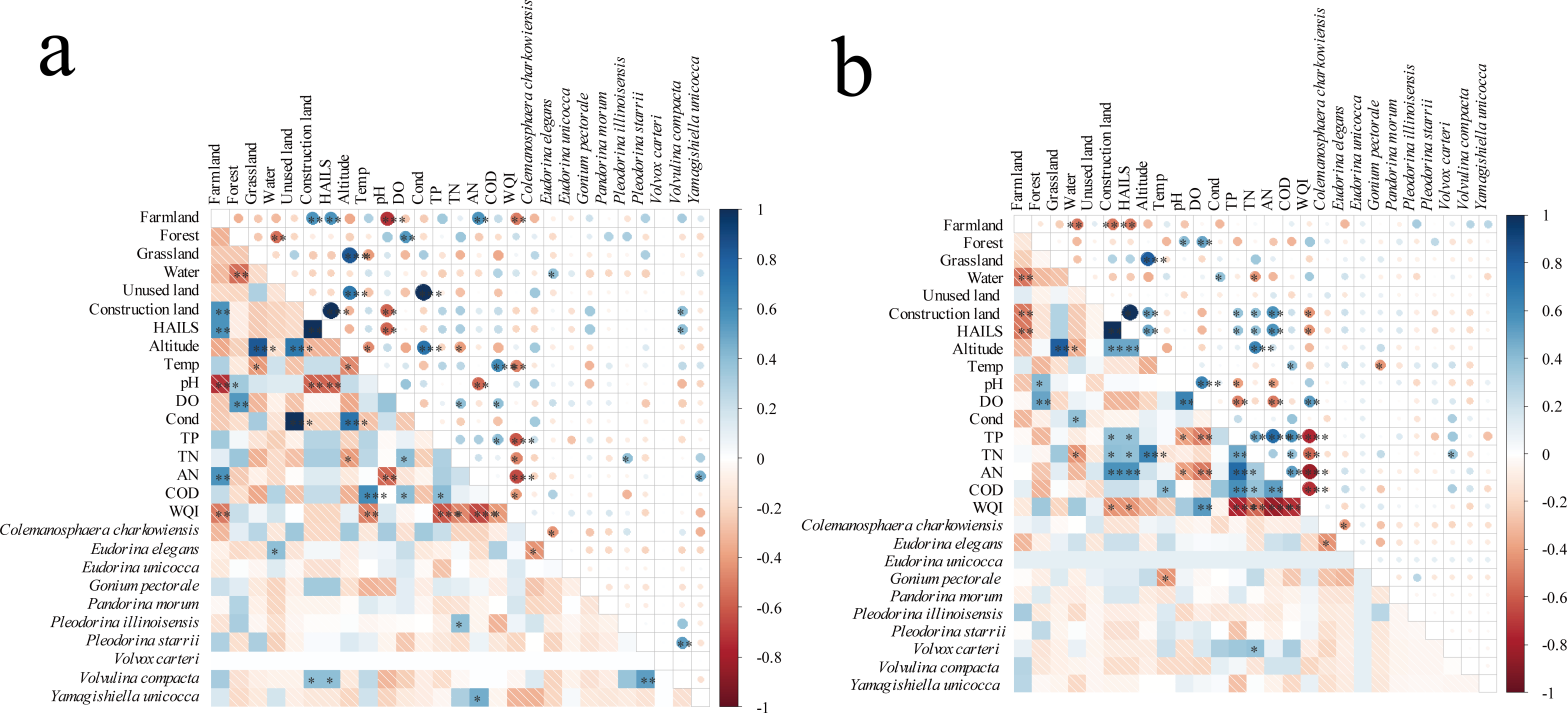
Spearman correlations between land-use types, water quality parameters, and CVA species are presented, with the lower left corner of the figure showing correlation coefficient magnitudes via color intensity, and the upper right corner using circle size and color to indicate these magnitudes. The color scale on the right provides a reference for the correlation coefficient values, ranging from −1 (strong negative correlation) to 1 (strong positive correlation). (**a**) Low HAILS. (**b**) High HAILS. ***P < 0.001, **P < 0.01, *P < 0.05.

Correlations between land-use type, water quality, and CVA community composition were further analyzed under varying HAILS levels. At low HAILS (Fig. 3a), *Pleodorina illinoisensis* positively correlated with TN, *Volvulina compacta* with construction land and HAILS, and *Yamagishiella unicocca* with AN. At high HAILS (Fig. 3b), *Volvox carteri* showed a significant positive correlation with TN, suggesting that the growth of these species is closely linked to nutrient levels.

**Fig 4:**
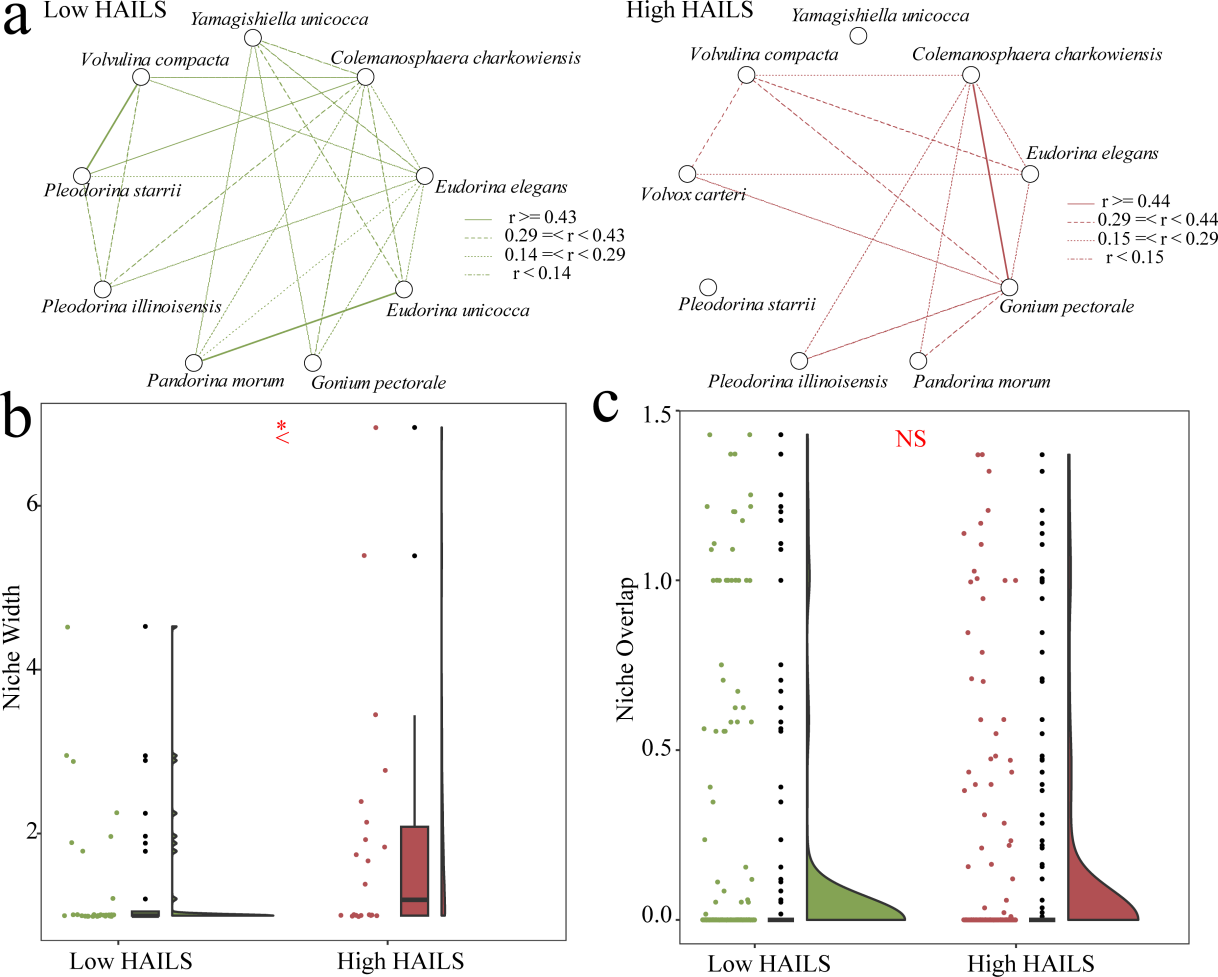
Niche width and overlap of CVA community. (**a**) The network graphs depict niche overlap patterns among CVA species under low and high HAILS. Each node represents a CVA species, while edges between nodes signify niche overlap indices. Different line styles (solid, dashed, dotted) correspond to distinct ranges of niche overlap values. Under low HAILS, the dense network structure indicates extensive niche overlap among species, reflecting a complex and interconnected community. In contrast, high HAILS simplify the network, with strong niche overlaps concentrated among specific species pairs. This suggests that environmental stress (HAILS) restructures niche relationships by filtering out weak interactions and promoting stronger overlaps among stress-tolerant taxa, thereby altering community assembly processes and ecosystem functionality. (**b, c**) Box plots of niche width and niche overlap between different HAILS. Box plot was in the middle, the scatters on the left side, and the half-violin whiskers on the right. *P < 0.05; NS, not significant. From low HAILS to high HAILS, the niche width of CVA species increases significantly, while there is no significant difference in niche overlap.

At both low and high HAILS levels (Fig. 3), *Eudorina elegans* negatively correlates with *Colemanosphaera charkowiensis*. Further analysis of species niche overlap consistently shows an ecological niche overlap between these two species (Fig. 4a). Under low HAILS, they significantly overlap with other CVA species, but this overlap decreases under high HAILS (Fig. 4a). The niche width for the CVA community widens from low to high HAILS (Fig. 4b), yet niche overlap remains consistent (Fig. 4c), suggesting that changes in niche width and overlap may relate to shifts in external environmental conditions.

### Impact of land-use intensification on CVA community assembly

**Fig 5:**
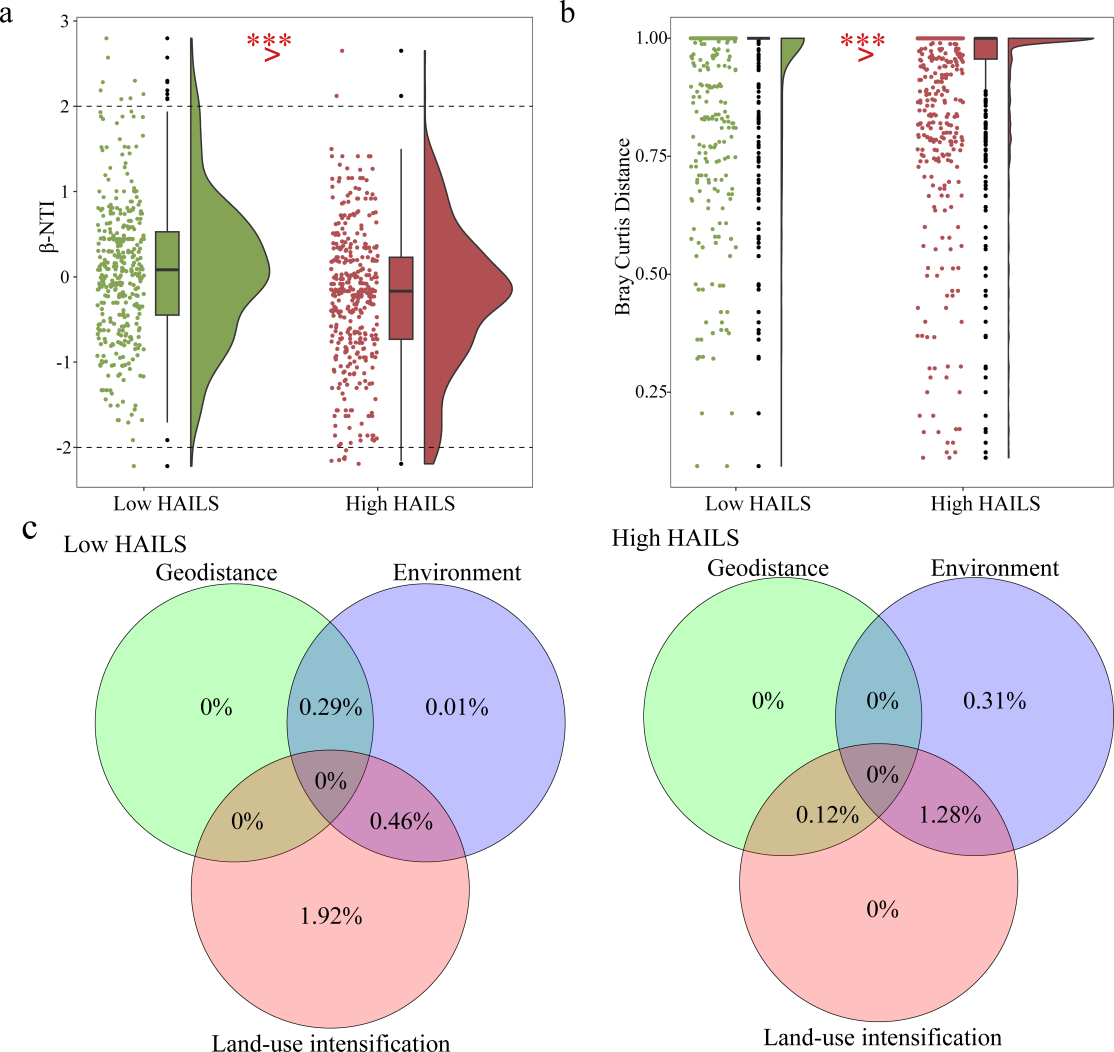
CVA community composition and assembly under different HAILS. (**a, b**) Box plots of β-NTI and Bray–Curtis distance between different HAILS. Box plot was in the middle, the scatters on the left side, and the half-violin whiskers on the right. ***P < 0.001. (**a**) β-NTI. (**b**) Bray–Curtis distance. (**c**) Venn diagram illustrating the relative contributions of geodistance, environment, and land-use intensification to explaining variance in ecological patterns under low HAILS and high HAILS. Each circle represents a distinct factor, with overlapping regions denoting joint contributions of factor combinations. Percentages within sections indicate the proportion of total variance uniquely or synergistically explained by the corresponding factor(s).

Our findings reveal that the β-NTI values of CVA communities mostly range between −2 and 2 (Fig. 5a), which indicates that stochastic processes predominate in community assembly. However, as HAILS levels increase from low to high, β-NTI values significantly decrease—specifically, the proportion of β-NTI values < −2 is 0.26% under low HAILS and 1.97% under high HAILS. This pattern suggests a shift toward homogeneous selection within CVA communities, accompanied by an increasing influence of deterministic processes on community assembly.

Using the Bray–Curtis distance index to measure β-diversity (higher values indicate greater dissimilarity and diversity), we found a significant decrease from low HAILS (0.9119 ± 0.2236) to high HAILS (0.8974 ± 0.2332), signaling reduced β-diversity (Fig. 5b). Both β-NTI and Bray–Curtis distance suggest community homogenization as HAILS increases. Analysis of the CVA assembly process shows an uptick in homogeneous selection and a downturn in heterogeneous selection with rising HAILS (Fig. S14), supporting this trend.

VPA analysis on the CVA community structure variance due to geodistance, environment, and land-use intensification indicates that from low to high HAILS, the explanatory power of the environment increases from 0.01% to 0.31%, while the combined effect of environment and land-use intensification rises from 0.46% to 1.28%. This highlights that intensifying human activities lead to structural changes in the CVA community, predominantly driven by enhanced environmental filtering (Fig. 5c).

**Fig 6:**
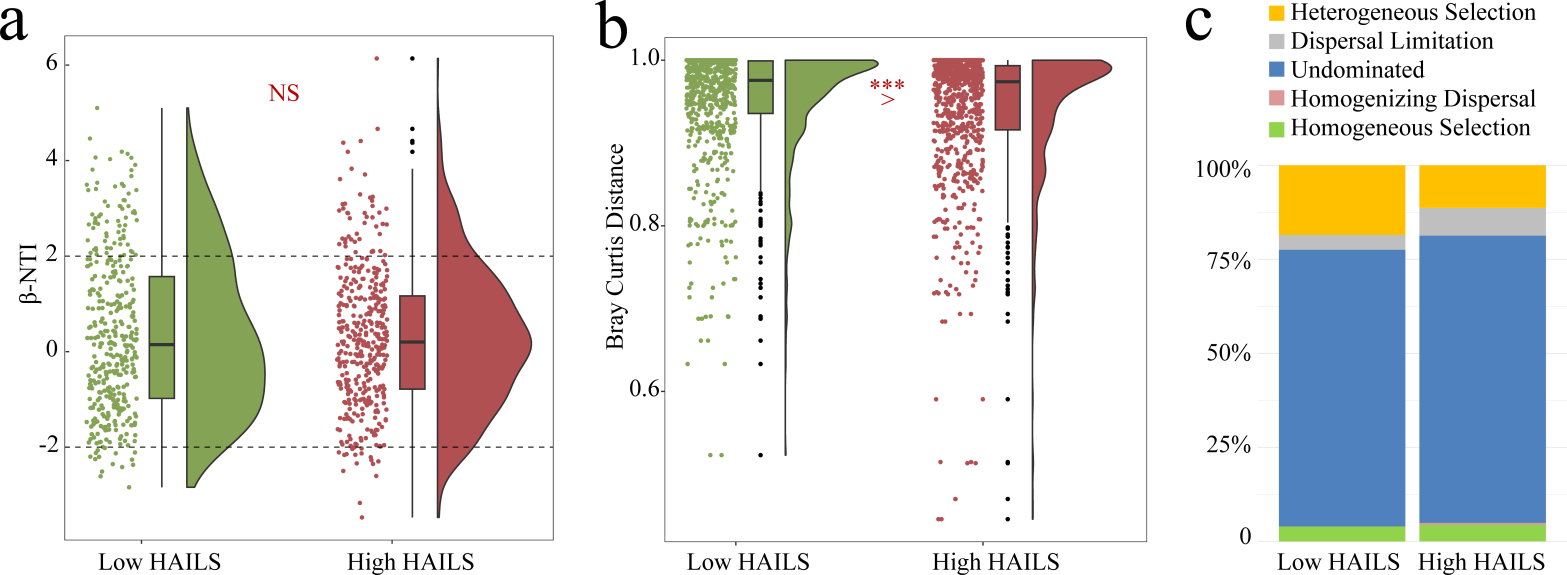
Eukaryotic community composition and assembly under different HAILS. (**a, b**) Box plots of β-NTI and Bray–Curtis distance between different HAILS. Box plot was in the middle, the scatters on the left side, and the half-violin whiskers on the right. ***P < 0.001. (**a**) β-NTI. (**b**) Bray-Curtis distance. (**c**) The different proportions of five ecological processes in eukaryotic community assembly under different HAILS.

Additional analysis of land-use intensification’s effect on other eukaryotic communities (Fig. S15) shows a significant rise in relative abundance for *Phascolodon* and *Epistylis* sp. from low to high HAILS, likely due to their adaptation to eutrophic conditions (40, 41). Like prokaryotes, eukaryotic community assembly is stochastically driven (Fig. 6a), but land-use intensification leads to reduced β-diversity (Fig. 6b), with a shift toward homogeneous selection over heterogeneous selection (Fig. 6c), echoing the trends seen in prokaryotic communities (17).

### Eutrophication causes CVA community homogenization

**Fig 7:**
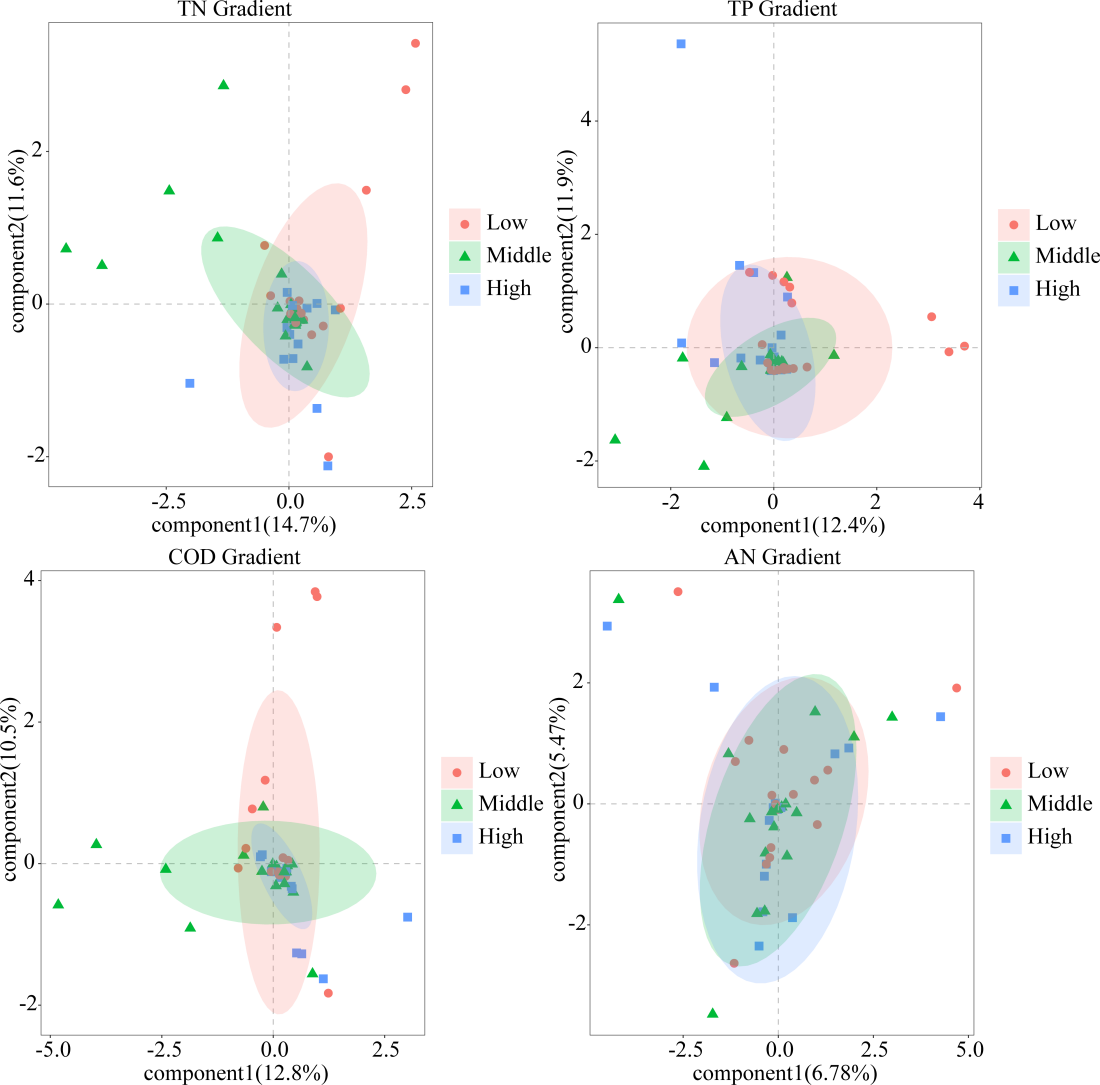
CVA community homogenization showed by PLS-DA along the trophic gradient (TN, TP, COD, AN).

NMDS analysis revealed distinct CVA communities under varied trophic conditions. Along the gradient of nutrients (TN, TP, COD, AN), taxonomic homogenization of CVA was evident (Fig. 7), with communities becoming increasingly clustered in highly eutrophic conditions, as measured by their proximity to the centroid.

SIMPER analysis identified key species contributing to CVA community disparities, revealing that under the influence of TN, TP, and COD, variations in CVA structure are primarily driven by *Colemanosphaera charkowiensis*, *Eudorina elegans*, and *Gonium pectorale* across different nutrient levels (Table S1).

## DISCUSSION

### Relationship between land use, water environment, and CVA community structure

To address the first two scientific questions proposed in the Introduction, we focus on how HAILS links to water environmental factors and further shapes the composition and niche characteristics of the CVA community. Our study indicates a significant negative correlation between HAILS and forests, suggesting that human activities are replacing natural landscapes (e.g., forest land) with construction land. This expansion contributes to increased non-point source pollution in the watershed, exacerbated by urbanization’s rise in construction land that facilitates the entry of debris, sediments, nutrients, and heavy metals into water bodies during storm runoff. Forest vegetation plays a crucial role in filtering these pollutants, which in turn enhances water quality and mitigates non-point source pollution (17, 22).

However, notably, as HAILS increases, the concentrations of TN, TP, AN, and COD in water also rise (Fig. 1b). This directly indicates a positive correlation between HAILS and water nutrient levels. Our study reveals that *Colemanosphaera charkowiensis*, *Yamagishiella unicocca*, and *Pleodorina starrii* are prevalent under lower nutrient conditions (low HAILS, Fig. 2a), while *Gonium pectorale*, *Eudorina elegans*, and *Volvox carteri* dominate in higher nutrient settings (high HAILS, Fig. 2a). *Volvox carteri*, exhibiting higher photosynthetic rates per carbon unit (42) and representing the largest CVA genus (4), favors nutrient-rich environments, is more abundant in high HAILS areas, and positively correlates with TN (Fig. 3b), consistent with the trend of larger phytoplankton thriving in eutrophic waters (43).

As HAILS increases, the ecological niche overlap among CVA species decreases (Fig. 4a), while the niche width of the community significantly expands (Fig. 4b). These changes are associated with elevated nutrient levels (Fig. 1b), which broaden the niche by increasing resource availability. Specifically, the eutrophication process (evidenced by increased TP, TN, and AN; Fig. 1b) provides more environmental resources for CVA. This may lead to shifts in their resource utilization strategies and adaptability to the environment, thereby widening the niche width of CVA. Changes in external environmental conditions could force species to develop new resource utilization strategies, potentially enhancing their adaptability and theoretically increasing genetic diversity (44). While these adaptive shifts raise the intriguing possibility of evolutionary diversification in the long term, such as through adaptive radiation (45), the current data indicate reduced β-diversity and community homogenization. Therefore, investigating whether and under what conditions elevated nutrients could ultimately promote adaptive radiation and increased biodiversity, despite initial homogenizing effects, represents a key direction for future research.

Our research revealed a negative correlation in abundance between *Eudorina elegans* and *Colemanosphaera charkowiensis*, both of which share high niche overlap under varying HAILS conditions (Fig. 4a). Morphologically similar, with traits differing mainly in contractile vacuoles and pyrenoids, both species reproduce through anisogamy (46). This likeness may equip them with comparable strategies against predators or environmental threats and similar nutritional needs (1). As Darwin pointed out in *The Origin of Species*, such similarity intensifies competition (47), a finding corroborated by studies linking morphological alikeness to parallel environmental demands (48). Interspecific competition is closely related to the ecological niche differentiation and overlap of species (49, 50). Intense competition among biologically similar forms prompts species to use resources more efficiently, potentially leading to coevolution, where interacting species influence each other’s evolution (51). To avoid fierce competition, species may differentiate their ecological niches, promoting an increase in biodiversity and further enhancing ecosystem complexity and stability (52).

### Homogenization of CVA communities due to land-use intensification changes

In this study, we investigate the mechanisms by which intensified land use drives CVA community homogenization, including shifts in community assembly processes and changes in β-diversity. The impact of land use on CVA and eukaryotic community assembly is similar to that on prokaryotes (17). Community assembly across different HAILS levels is chiefly influenced by stochastic processes (Fig. 5), which also clarifies why increased biological complexity within the CVA does not guarantee wider distribution or higher abundance (Fig. 2b). Moreover, an organism’s adaptation to its environment hinges on multiple factors, and there comes a point where further complexity becomes detrimental due to the cost (6). Therefore, higher complexity does not necessarily imply greater adaptability. Given the current data set focused on the Yangtze River Basin, it is premature to completely dismiss the connection between complexity and adaptability. On the contrary, the observed pattern that complexity does not directly translate into ecological dominance is an intriguing hypothesis worth further exploration. Future research needs to expand the sampling scope, especially for groups such as CVA taxa with limited distribution records. This will be crucial for clarifying whether this phenomenon represents a universal ecological rule or a context-specific occurrence.

Intensified land use reduces β-diversity and promotes community similarity, consistent with research linking land use to biodiversity homogenization (53–55). High HAILS correlate with increased nutrient levels, favoring eutrophic-adapted species and driving community convergence through environmental filtering, which shapes community membership by selecting species suited to specific conditions (56–59). This process, linked to community homogenization, is evident in the relationship between diatom β-diversity and environmental heterogeneity (60). In this study, certain CVA species (such as *Eudorina elegans*, *Gonium pectorale*, *Volvox carteri*) exhibited higher relative abundance under high HAILS (Fig. 2a), suggesting their susceptibility to strong environmental filtering by factors, such as TN, TP, and COD (Fig. 1b and 7). Notably, *Volvox carteri*—a representative of these pollution-tolerant species—owes its ability to tolerate pollution and survive in such stressful environments to key biological traits: as the largest CVA genus with higher photosynthetic rates per carbon unit, it can efficiently capture and utilize the elevated nutrients (e.g., TN) in high HAILS habitats. This trait-based resource utilization advantage directly supports its dominance under polluted, nutrient-rich conditions.

High HAILS correlate with expansive construction land, which is intrinsic to urbanization and a key driver of biological homogenization (61). Urban sprawl typically causes habitat fragmentation and environmental decay, favoring certain species’ proliferation. Cities often share similar environments, leading to analogous adaptive species. Studies on urban plant composition have shown that it is more alike across cities than in natural areas, maintaining similarity despite increasing distances (61). Analysis across 58 counties confirmed this, revealing greater similarity in highly urbanized county plant life compared to less urbanized ones (62). Biological homogenization simplifies food webs, increasing ecosystem vulnerability and reducing species dispersal (63). This uniformity causes communities to respond similarly to ecological disturbances, diminishing regional barrier effects (64). Loss of β-diversity due to species homogenization depletes spatial community diversity, hindering speciation (63). To counteract this homogenization, restoring ecosystems through reforestation and implementing robust land-use planning to safeguard biodiversity and support sustainable development are recommended.

### Conclusions

This study focuses on CVA, a model group for evolutionary research, and for the first time examines how human land-use intensity shapes CVA community dynamics, filling a critical research gap. Our study demonstrated that increased intensity of land use leads to elevated nutrient levels in water bodies, which in turn affects the community structure of CVA. For instance, high HAILS result in a broader ecological niche for CVA, favoring larger-bodied or pollution-tolerant CVA species. The community assembly of CVA resembles that of eukaryotes and prokaryotes, where stochastic processes play a decisive role. However, as HAILS increases, nutrient levels rise in the water, enhancing the contribution of environmental filtering to the assembly of CVA communities. Notably, high HAILS can lead to homogenization of CVA community structures, potentially causing a decrease in biodiversity, simplification of food webs, and an increase in ecosystem fragility. The findings of this study provide new insights into how eukaryotic communities respond to anthropogenic pressures and how land-use changes reshape the structure and assembly mechanisms of freshwater ecosystems. Meanwhile, recommendations are put forward to improve land-use planning and management for maintaining the diversity of CVA communities.

## Data Availability

The data that support the findings of this study are openly available at the sequence read archive (http://www.ncbi.nlm.nih.gov/sra) at the National Center for Biotechnology Information under accession no. PRJNA1110346.

## SUPPLEMENTAL MATERIAL

Supplemental material spectrum.02174-25-s0001.docx Figures S1 to S15; Table S1.

ASM does not own the copyrights to Supplemental Material that may be linked to, or accessed through, an article. The authors have granted ASM a non-exclusive, world-wide license to publish the Supplemental Material files. Please contact the corresponding author directly for reuse.
