# Supplementary material for: The impact of land-use intensity on the community dynamics of colonial volvocine algae in the Yangtze River Basin
Source: Microbiol Spectr. 2025 Dec 17;14(2):e02174-25. doi: 10.1128/spectrum.02174-25 (PMC12889134; doi:10.1128/spectrum.02174-25)
Supplement: Supplemental material — Figures S1 to S15; Table S1. [file spectrum.02174-25-s0001.docx]

**The Impact of Land Use Intensity on the Community Dynamics of Colonial Volvocine Algae in the Yangtze River Basin**

Yuxin Hu^1,2†^, Jiwei Zhang^1,2†^, JingJing Lin^1,2^, Xiaolong Huang^1,2*^, Jie Huang^1,2*^

^1^ Changjiang Basin Ecology and Environment Monitoring and Scientific Research Center, Changjiang Basin Ecology and Environment Administration, Ministry of Ecology and Environment, Wuhan 430010, Hubei, China

^2^ Hubei Provincial Key Laboratory for Basin Ecology Intelligent Monitoring-Prediction and Protection, Wuhan, 430010

* Correspondence:

Xiaolong Huang, h.xl0210@163.com

Jie Huang, huangjie@cjjg.mee.gov.cn

† These authors contributed equally to this work.


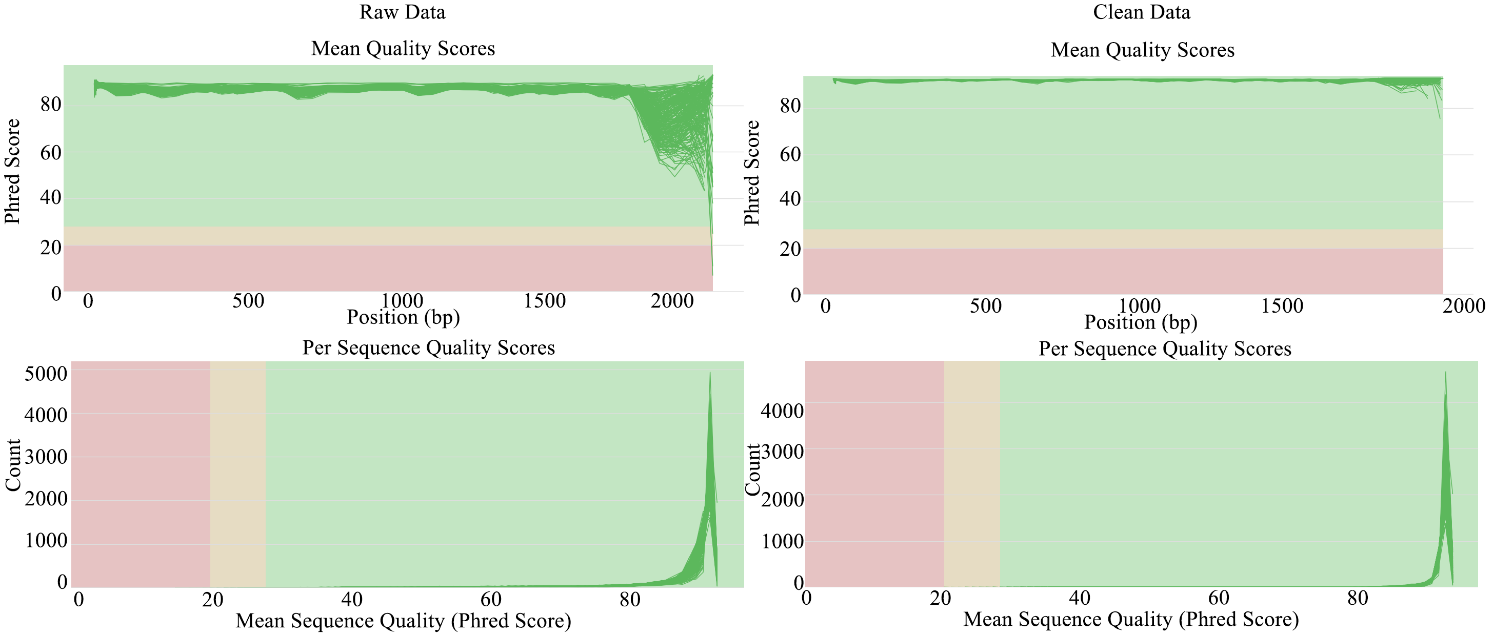


**Supplementary Figure 1**. Quality control of raw data. Upper row: the mean quality value across each base position in the read. Lower row: the number of reads with average quality scores. Left column: raw data. Right column: clean data.





**Supplementary Figure 2**. Phylogenetic analysis of *Colemanosphaera charkowiensis* based on 18S ribosomal RNA gene





**Supplementary Figure 3**. Phylogenetic analysis of *Gonium* based on 18S ribosomal RNA gene.





**Supplementary Figure 4**. Phylogenetic analysis of *Eudorina* based on 18S ribosomal RNA gene.





**Supplementary Figure 5**. Phylogenetic analysis of *Yamagishiella* based on 18S ribosomal RNA gene.





**Supplementary Figure 6**. Phylogenetic analysis of *Pleodorina* based on 18S ribosomal RNA gene.





**Supplementary Figure 7**. Phylogenetic analysis of *Volvulina* based on 18S ribosomal RNA gene.





**Supplementary Figure 8**. Phylogenetic analysis of *Pandorina* based on 18S ribosomal RNA gene.





**Supplementary Figure 9**. Phylogenetic analysis of *Volvox* based on 18S ribosomal RNA gene.


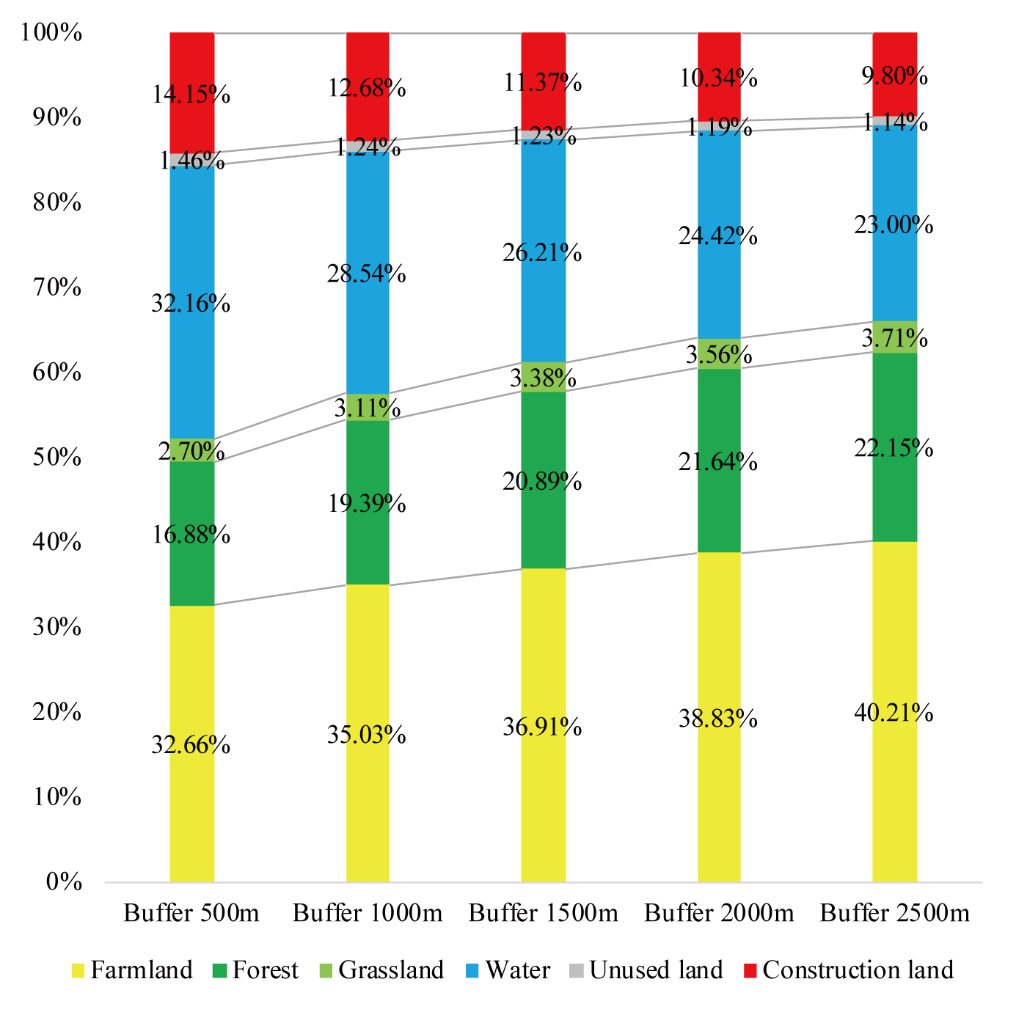


**Supplementary Figure 10.** The proportional area of different land use types under different buffer zones.


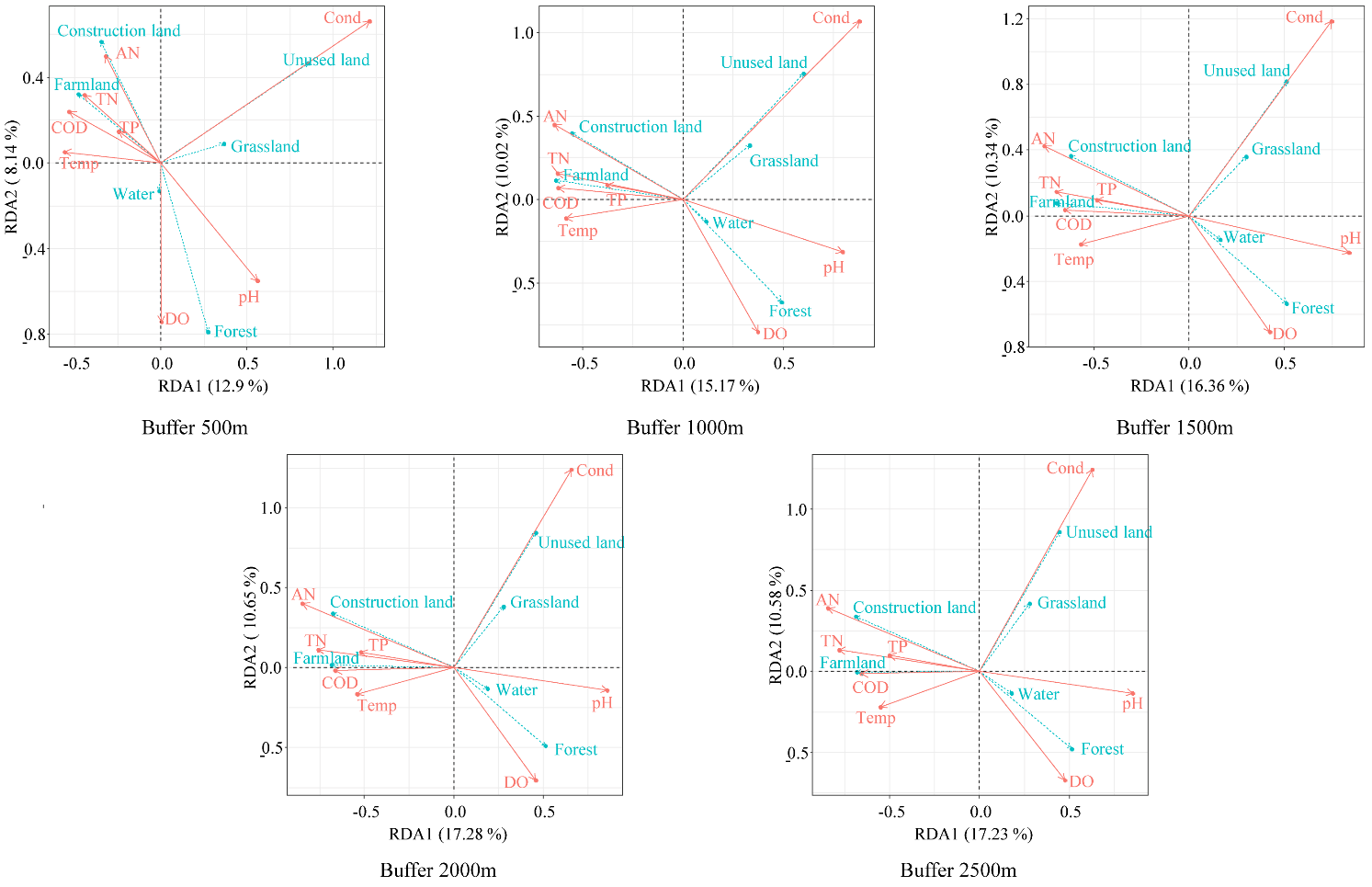
 **Supplementary Figure 11.** RDA analysis of water quality parameters and land use types in different buffer zones.

**
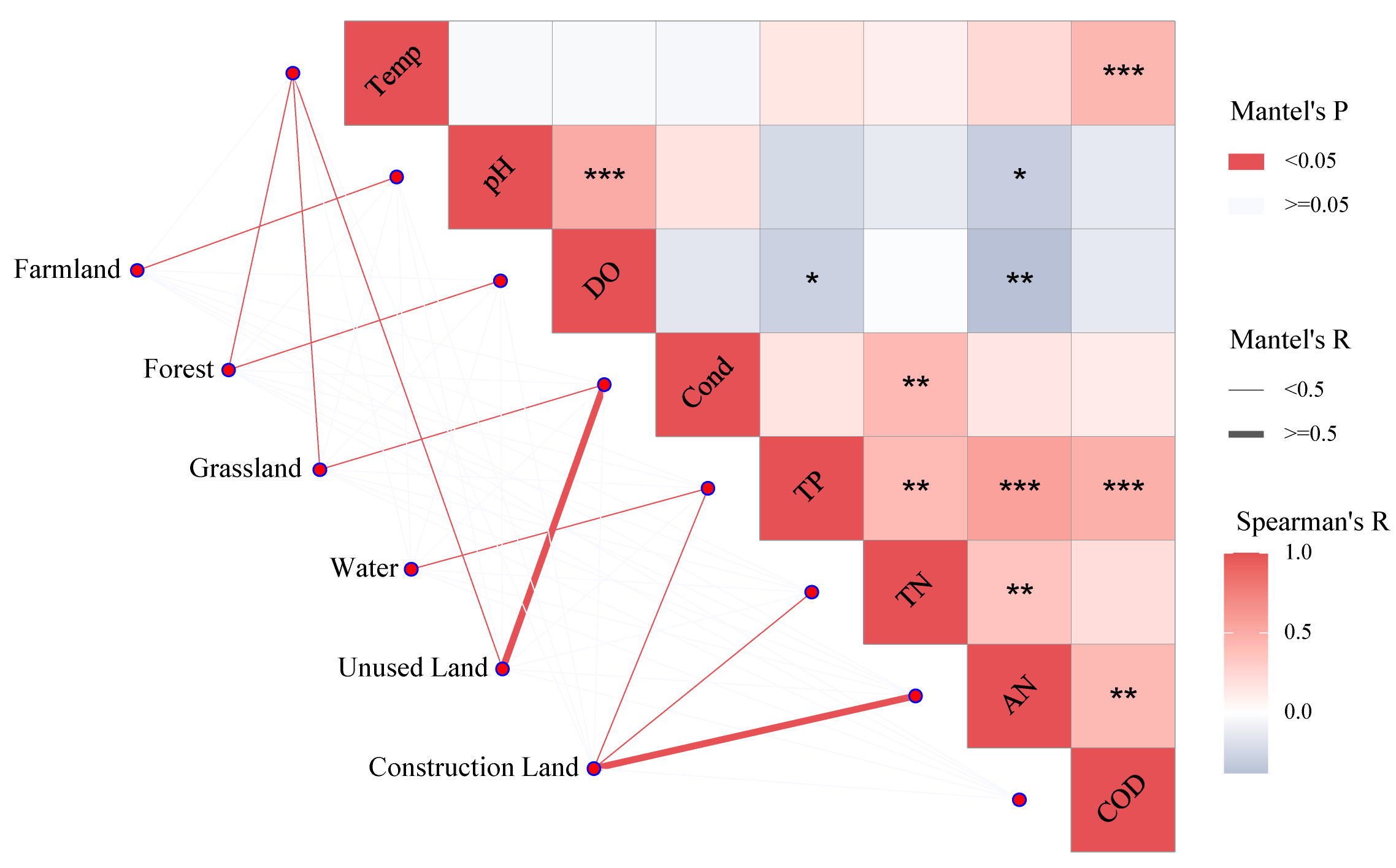
**

**Supplementary Figure 12.** The relationship between various land use categories and water quality indicators was examined using the Mantel test, within a 2 km buffer.


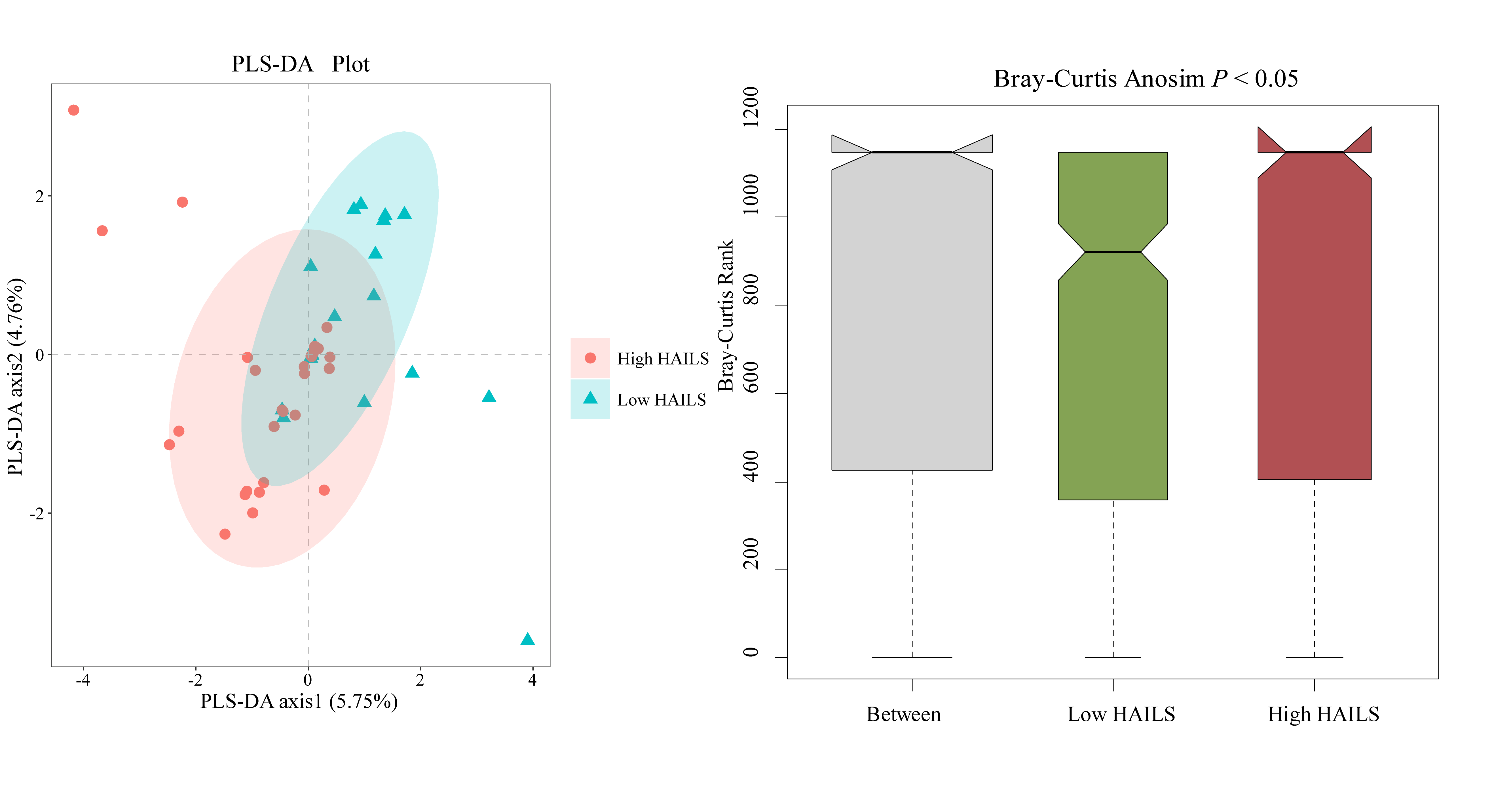


**Supplementary Figure 13.** The left figure shows the PLS-DA analysis of different HAILS sites, and the right figure shows the ANOSIM analysis of different HAILS sites.


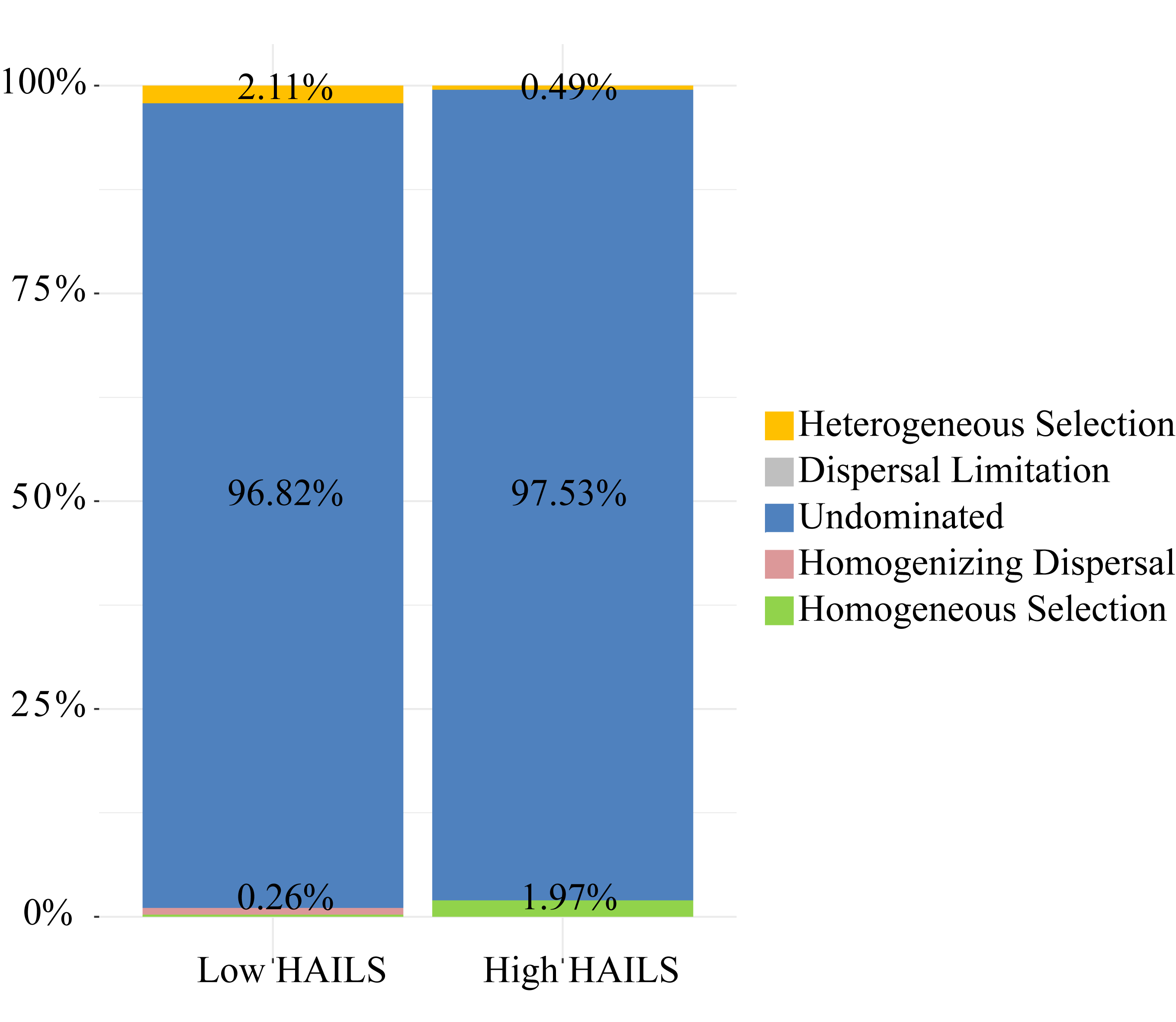


**Supplementary Figure 14.** The different proportions of five ecological processes in CVA community assembly under different HAILS.


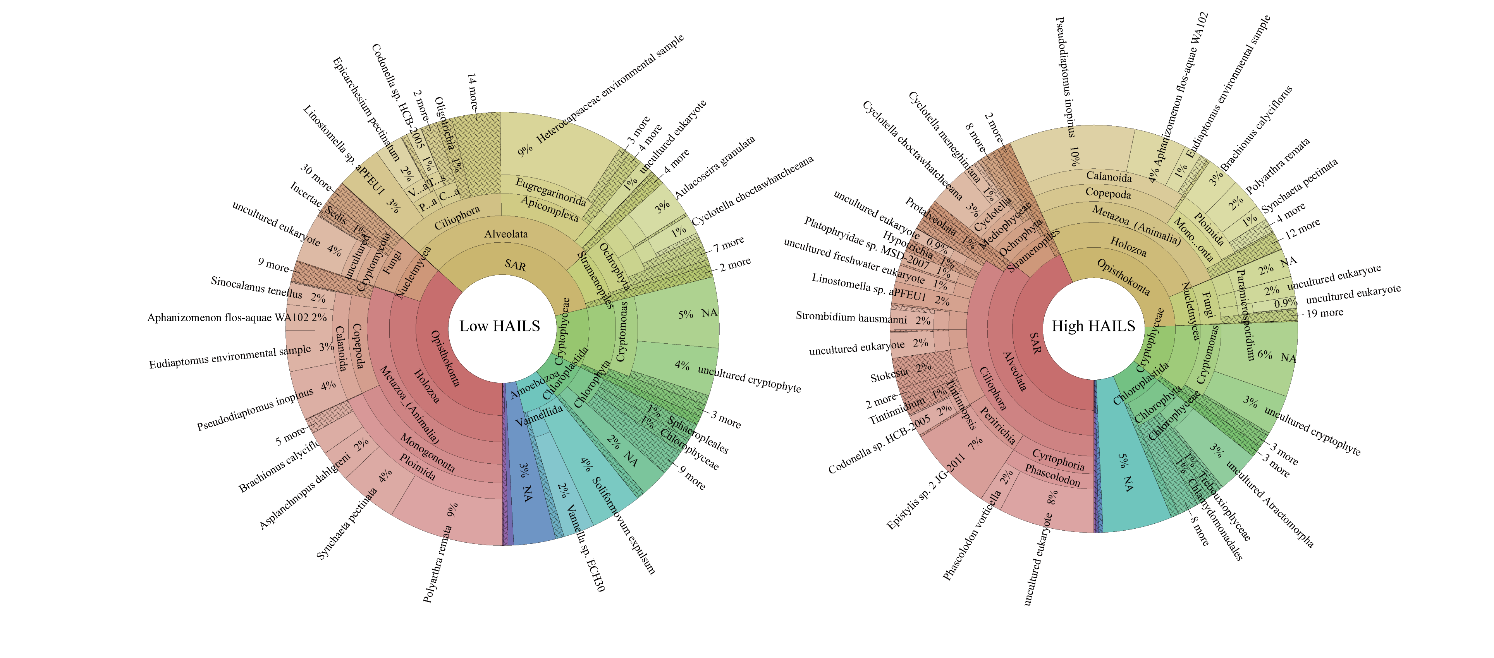


**Supplementary Figure 15.** Krona plot of eukaryotes community composition under different HAILS.

**Supplementary Table 1.** The contributions of characteristic species under different trophic gradients identified by SIMPER procedure. Divide the nutrient concentration into three equal parts, low, medium, and high.

|  | High vs Low | | | High vs Middle | | | Middle vs Low | | |
| --- | --- | --- | --- | --- | --- | --- | --- | --- | --- |
|  | COD | TN | TP | COD | TN | TP | COD | TN | TP |
| *Colemanosphaera charkowiensis* | 0.239714453 | 0.241108 | 0.251814 | 0.268218 | 0.329345 | 0.308475 | 0.288019 | 0.229677 | 0.232379 |
| *Eudorina elegans* | 0.192459431 | 0.171521 | 0.225082 | 0.195687 | 0.197926 | 0.133062 | 0.204593 | 0.220813 | 0.256816 |
| *Gonium pectorale* | 0.213140401 | 0.221788 | 0.200948 | 0.239088 | 0.174145 | 0.216462 | 0.149721 | 0.200125 | 0.178556 |
| *Eudorina unicocca* | 0.003234775 | 0 | 0.003021 | 0 | 0.003132 | 0 | 0.002835 | 0.003025 | 0.003385 |
| *Pandorina morum* | 0.042090391 | 0.048586 | 0.027875 | 0.04027 | 0.042476 | 0.062463 | 0.043498 | 0.034899 | 0.031734 |
| *Pleodorina illinoisensis* | 0.018294408 | 0.001936 | 0.009945 | 0.008259 | 0.018636 | 0.016573 | 0.00988 | 0.016717 | 0.009778 |
| *Pleodorina starrii* | 0.051073687 | 0.037552 | 0.051684 | 0.03131 | 0.025814 | 0.04016 | 0.036111 | 0.058001 | 0.031451 |
| *Volvox carteri* | 0 | 0.038829 | 0.036214 | 0.040014 | 0 | 0.012434 | 0.03837 | 0.037352 | 0.029444 |
| *Volvulina compacta* | 0.030057263 | 0.033115 | 0.00828 | 0.014266 | 0.030067 | 0.037086 | 0.031359 | 0.012218 | 0.027662 |
| *Yamagishiella unicocca* | 0.080646191 | 0.091696 | 0.074721 | 0.031081 | 0.025773 | 0.031154 | 0.084346 | 0.079094 | 0.091873 |
